# Evaluation of hernia surgical meshes sterilised with ethylene oxide for adoption under UK regulations

**DOI:** 10.1007/s00464-023-10460-9

**Published:** 2023-09-20

**Authors:** Alessandra Grillo, Zargham Hyder, Vivek Mudera, Alvena Kureshi

**Affiliations:** 1 Centre for 3D Models of Health and Disease, Division of Surgery & Interventional Science, UCL, London, UK; 2 Hydermed Limited, Woodford Green, UK; 3 Homerton University Hospital, NHS Trust, London, UK

**Keywords:** Hernia repair, Mosquito mesh, Frugal innovation, Ethylene oxide, Sterilisation

## Abstract

**Background:**

Low-cost meshes (LCM) have been successfully used in low-income countries (LIC) over the past decades, demonstrating comparable surgical outcomes to commercial meshes at a fraction of the cost. However, LIC sterilisation standards (autoclave sterilisation at 121 °C) do not meet UK regulations for medical devices, which require either ethylene oxide (EO) sterilisation or steam sterilisation at 134 °C. Therefore, the aim of this study was to sterilise UK LCM and characterise their mechanical properties and in vitro biocompatibility to verify whether EO sterilisation causes changes in the mechanical properties and biocompatibility of LCM.

**Methods:**

EO sterilised LCM were used. Uniaxial tensile tests were performed to measure mechanical properties. Biocompatibility was measured through viability and morphology of Human Dermal Fibroblasts (HDFs) cultured in mesh-conditioned media, and by calculating the metabolic activity and proliferation of HDFs attached on the meshes, with alamarBlue assay.

**Results:**

Break stress of LCM1 was significantly higher than LCM2 (*p* < 0.0001), while Young’s modulus of LCM1 was significantly lower than LCM2 (*p* < 0.05) and there was no significant difference in break strain. Viability and morphology showed no significant difference between LCM and control. Attachment and proliferation of HDFs on LCM showed a better proliferation on LCM2 than LCM1, with values similar to the control at the final time point.

**Conclusions:**

We demonstrated that EO sterilisation affects LCM mechanical properties, but they still have values closer to the native tissues than the commercially available ones. We also showed that in vitro biocompatibility of LCM2 is not affected by EO sterilisation, as HDFs attached and proliferated on the mesh, while EO affected attachment on LCM1. A more detailed cost analysis of the potential savings for healthcare systems around the world needs to be performed to strengthen the cost-effectiveness of this frugal innovation.

Over the past decades, it has become more common for surgeons operating in low-income countries (LIC) to re-purpose “mosquito nets” for hernia repair surgery, particularly in countries where hernias are extremely prevalent and commercial meshes (CM) too expensive and therefore not affordable [1–3]. In the late nineties, the first multi-centre clinical trial was conducted in India by Tongaonkar et al. who implanted a low-cost mesh made of a mixture of polypropylene and polyethylene and analysed the postoperative outcomes up to 5 years after the surgery [4]. The results from this trial reported that the low-cost meshes (LCM) adopted had properties that were comparable to the commonly used CM but had a significantly reduced cost, reportedly as much as 10,000 times cheaper than CM [5]. Importantly, recurrences and infections significantly dropped following hernia repairs with LCM [4]. Following this case, other surgeons adopted the cheaper LCM to repair hernias in Ghana, Burkina Faso, Cameroon and India [2, 6–8]. Numerous studies involving surgical repair of open inguinal hernias with LCM reported encouraging short-term and long-term surgical outcomes, as they did not cause increased inflammation, infections or recurrence compared to CM [9].

Moreover, the logic to reinforce the abdominal wall by synthetic mesh, hence forming stronger scar plates became popular among the surgical community in the late nineties. The best mesh was considered to be the one with strong material to induce most fibrosis and be able to withstand physiological intra-abdominal pressure surges [10]. Studies on mesh functions proved that the tensile strength of a mesh required to withstand the maximum abdominal pressure is only a tenth of that of most meshes and in fact, CM are over-engineered for their purpose [11, 12]. This realisation led to a move towards the use of large pore and lightweight mesh.

Porosity of mesh determines the inflammatory reaction within the tissues. Pore size of 75 µm and above is adequate for the inflammatory process and tissue ingrowth in order to have a flexible scar plate.

Mesh with the large pore (> 1 mm) has small surface area, therefore light in weight typically 35 g/m^2^. Lightweight meshes have acceptable tensile strength and are cost effective [13]. Studies on tensile strength have shown a maximum rise in intra-abdominal pressure during coughing and jumping (170 mmHg), therefore meshes required to withstand at least 180 mmHg of pressure within the intra-abdominal compartments, equates to tensile strength of 32 N/cm [14]. Commercially available lightweight mesh has desired tensile strength, and therefore widely employed in clinical practice. LCM has also shown similar biomechanical properties as native human tissues in various studies, however, poor strength of these studies led to low acceptance rates in clinical practice within the European hemisphere.

Additionally, despite the affordable costs of LCM compared to the CM and the promising post-surgical outcomes, there is still scepticism around the use of LCM in the UK and other developed countries. One barrier preventing its adoption is the sterilisation method of meshes. In the developing world, meshes are typically sterilised using high temperatures up to 134 °C. Stephenson and Kingsnorth [9] compared meshes sterilised at 121 °C and 134 °C and concluded that at higher temperature the texture of the mesh changed and became stiffer and more difficult to handle. At 121 °C, the material properties were not adversely affected but it carries a risk of transmission of spongiform encephalopathies. In the UK, and in other developed countries, there are strict guidelines around sterility of medical devices and temperatures lower than 134 °C are not permitted. A common method of sterilisation used for most CM is ethylene oxide (EO). This is a well-established method used for sterilisation of medical devices which uses a lower temperature compared to steam sterilisation and ensures the full destruction of bacteria, particularly Gram-positive bacteria [9]. Since this method takes a longer amount of time and is more expensive to perform than steam sterilisation, it is not a viable option in LIC [9, 15]. However, when compared to CM, EO sterilisation costs would be still cheaper and could represent a valuable cost-effective alternative.

This study aims to provide in vitro characterisation of LCMs following EO sterilisation to evaluate mesh efficacy in terms of biocompatibility and material properties. This will demonstrate whether or not EO sterilisation could be used on LCMs in the UK to satisfy regulations, and thus be adopted for use in the UK and other high-income countries.

## Materials and methods

The mosquito meshes (named LCM) were purchased from UK retailers without pre-impregnation nor anti-insecticides. LCM1 (Mountain Warehouse, UK) and LCM2 (Purple Turtle, UK) are made from nylon and polyester, respectively.

### EO sterilisation

The LCM were pre-cut according to experimental needs and sealed into sterilising pouches, before being processed with EO gas by Andersen Products Ltd (UK). The process involved a precondition step at a minimum of 47̇ °C for 1 h 30 m, exposure to EO gas for 12 h at 29.5 °C followed by a purge time of 2 h at 29.1 °C. Lastly, the pouches were left to ventilate at room temperature for 48 h before use.

### Mechanical tests

EO LCM1 and LCM2 meshes (*N* = 10 and *N* = 12, respectively) were cut into dog-bone shapes (width: 10 mm; grip-to-grip distance: 20 mm). Thickness of the samples was measured prior to the tests using a thickness gauge (Mitutuyo 543-402BS, Sakado, Japan) with a resolution of 0.01 mm. Uniaxial tensile tests (BT1-FR5.0TN, Zwick Roell Group, Ulm, Germany) with a 0.5 kN loading cell (KAP-TC, Zwick Roell Group, Ulm, Germany) were performed in displacement-controlled mode (8 mm/min) until breaking point, excluding samples not breaking at their centre. Break stress was calculated as the maximum force divided by the grip-to-grip distance (N/cm). Break strain was expressed as the value of strain at maximum displacement (% of extension). Young’s modulus was calculated as the gradient of the stress–strain curve and selecting the slope of the linear region (N/cm).

### Cell culture

Human adult-donor Dermal Fibroblasts (HDF) were kindly gifted by Prof. Umber Cheema and originally purchased through Promocell (Heidelberg, Germany). They were cultured in growing media made of high glucose DMEM supplemented with 10% FBS and 1% Pen/Strep at 37 °C, 5% CO_2_ and sub-cultured upon reaching 80% confluence, using the trypsinisation method. Human dermal fibroblasts (HDFs) were used for experiments at passage 7–9 and resulted free from the presence of mycoplasma.

### Seeding efficiency on meshes

Seeding efficiency on the meshes was calculated as percentage of cells attached from the initial seeding density and was of 5% and 10% for LCM1 and LCM2, respectively. Therefore, the number of cells to be seeded was chosen considering the efficiency of attachment in order to have the same number of cells at time 0 for both meshes.

### Immunofluorescence

Samples were fixed in 4% Paraformaldehyde (PFA) for 20 min, washed three times in PBS and permeabilised with a 0.25% Triton X-100 (Sigma-Aldrich) solution. After three further washes in PBS, samples were incubated with 1:1000 Phalloidin TRITC (Sigma-Aldrich, UK) for 1 h to visualise cells’ actin filaments and then mounted on glass microscope slides with VECTASHIELD® DAPI (Vector Laboratories Inc.) for nuclei visualisation. Samples were imaged with Zeiss AxioObserver Microscope with ApoTome.2 feature and Zeiss ZEN software (Zeiss, Oberkochen, Germany).

### Cytotoxicity test—cell viability and morphology

Mesh-conditioned media was prepared to evaluate cytotoxicity (according to the ISO 10993-5 standard). In particular, a rectangular piece of each mesh (300 cm^2^) previously cleaned with 70% ethanol and phosphate buffer solution was soaked in 50 ml of growing media for 24 h at 37 °C. Once the meshes were removed, the mesh-conditioned media obtained were then used to culture HDFs for 72 h at 37 °C, 5% CO_2_ and evaluate viability and morphology.

Percentage of viable cells was assessed by adding 500 μl of LIVE/DEAD® Viability/Cytotoxicity Assay kit per well according to the manufacturer's protocol (Thermo Fisher Scientific—Life Technologies), where Calcein-AM (green) indicates live cells while ethidium homodimer (EthD-1) (red) indicates cell death. Cells cultured in growth media represented negative control (CTRL−) and 70% methanol positive control or dead cells (CTRL+).

For morphology assessments, Images were taken with Zeiss AxioObserver Microscope and Zeiss ZEN software (Zeiss, Oberkochen, Germany) and analysed using the image processing program ImageJ (NIH, USA). Actin filaments of HDFs were stained with 1:1000 Phalloidin FITC (Sigma-Aldrich, UK) and nuclei were visualised using VECTASHIELD® DAPI mounting media (Vector Laboratories Inc.) on glass microscope slides. Area and cell elongation (short/long axis ratio) were calculated using the image processing program ImageJ (NIH, USA).

### Alamar Blue—metabolic activity assay

Proliferation of HDFs cultured on the two meshes was calculated through the metabolic activity measured with alamarBlue™ assay (Invitrogen, Thermo Fisher, Netherlands). The working solution was prepared as per the manufacturer’s protocol, using a 1:9 ratio of alamarBlue™ reagent to growing media in a sterile environment and adding 1 ml of solution to each sample, incubating for 4 h at 37 °C, 5% CO_2_. The solution was transferred in triplicates (100 μl from each sample) to a 96-well plate and the plate was scanned with a CLARIOstar® microplate reader (BMG LABTECH GmbH, Germany) in fluorescence mode (excitation 560 nm, emission 590 nm). A series of ascending cell densities was seeded and the metabolic activity measured to produce a standard curve. Unknown values were then interpolated from the curve with GraphPad Prism to infer cellular proliferation (version 9.3.1 for Windows, GraphPad Software, San Diego, California USA, www.graphpad.com).

### Statistical analysis

Mechanical properties, viability, cell area and cell elongation data were analysed with Welch’s *t* test. Metabolic activity values were analysed using Brown-Forsythe and Welch ANOVA tests with Dunnett’s correction for multiple comparisons. All data were represented as mean ± SD. All tests were conducted with *N* = 3, unless stated otherwise.

## Results

**Fig. 1:**
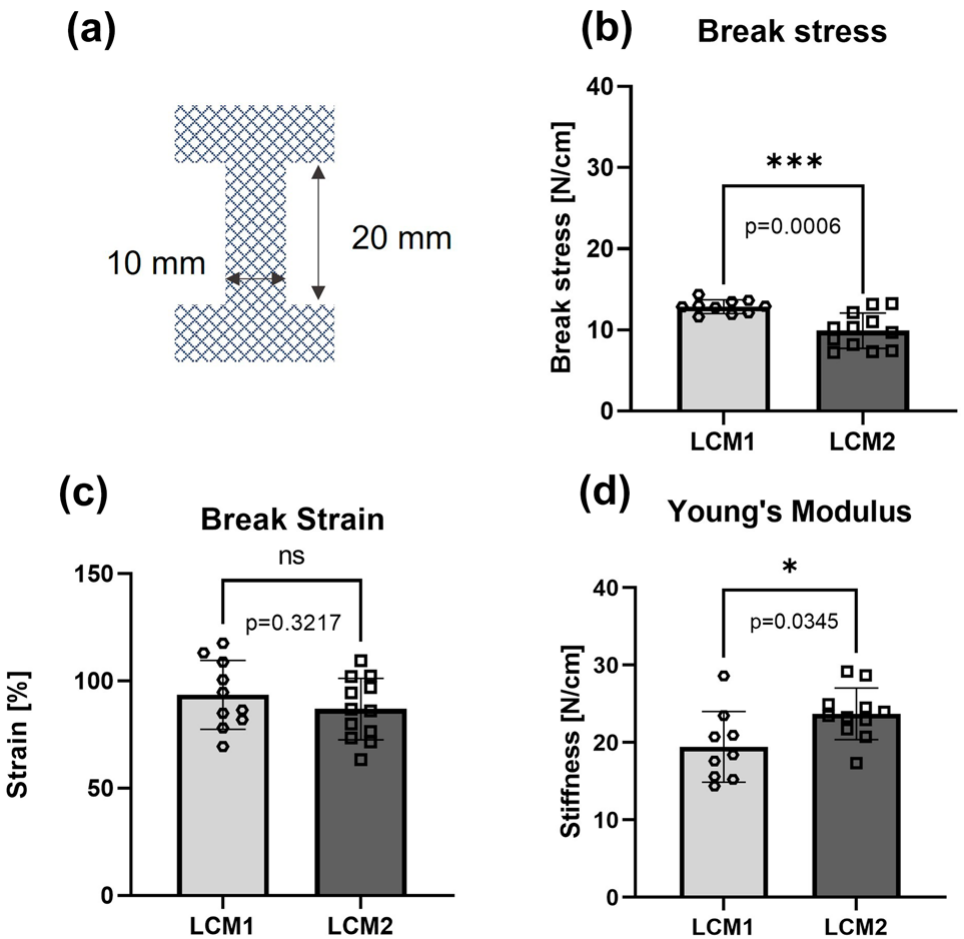
Mechanical properties of EO meshes. **a** Dimensions of mesh samples for mechanical testing. **b** Break stress, **c** break strain and **d** Young’s modulus of EO LCM1 and EO LCM2. *N* = 10 and *N* = 12 for LCM1 and LCM2, respectively. *p < 0.05, ***p < 0.001

Break stress, break strain and Young’s modulus of EO LCM1 and EO LCM2 were obtained from uniaxial tensile tests. Values of break stress for EO-sterilised LCM1 were 12.9 ± 0.9 N/cm compared to 9.9 ± 2.1 N/cm for EO-sterilised LCM2, with a significant difference between the two (*p* < 0.001) (Fig. 1b). Break strain was not significantly different between the two treated meshes, as LCM1 and LCM2 had extensibility values of 93.6 ± 15.2% and 86.9 ± 13.7%, respectively (Fig. 1c). For Young’s modulus measurements, EO-treated LCM2 was significantly stiffer than EO-treated LCM1, with values of 19.4 ± 4.1 N/cm and 23.7 ± 4.3 N/cm, respectively (*p* < 0.05) (Fig. 1d).

**Fig. 2:**
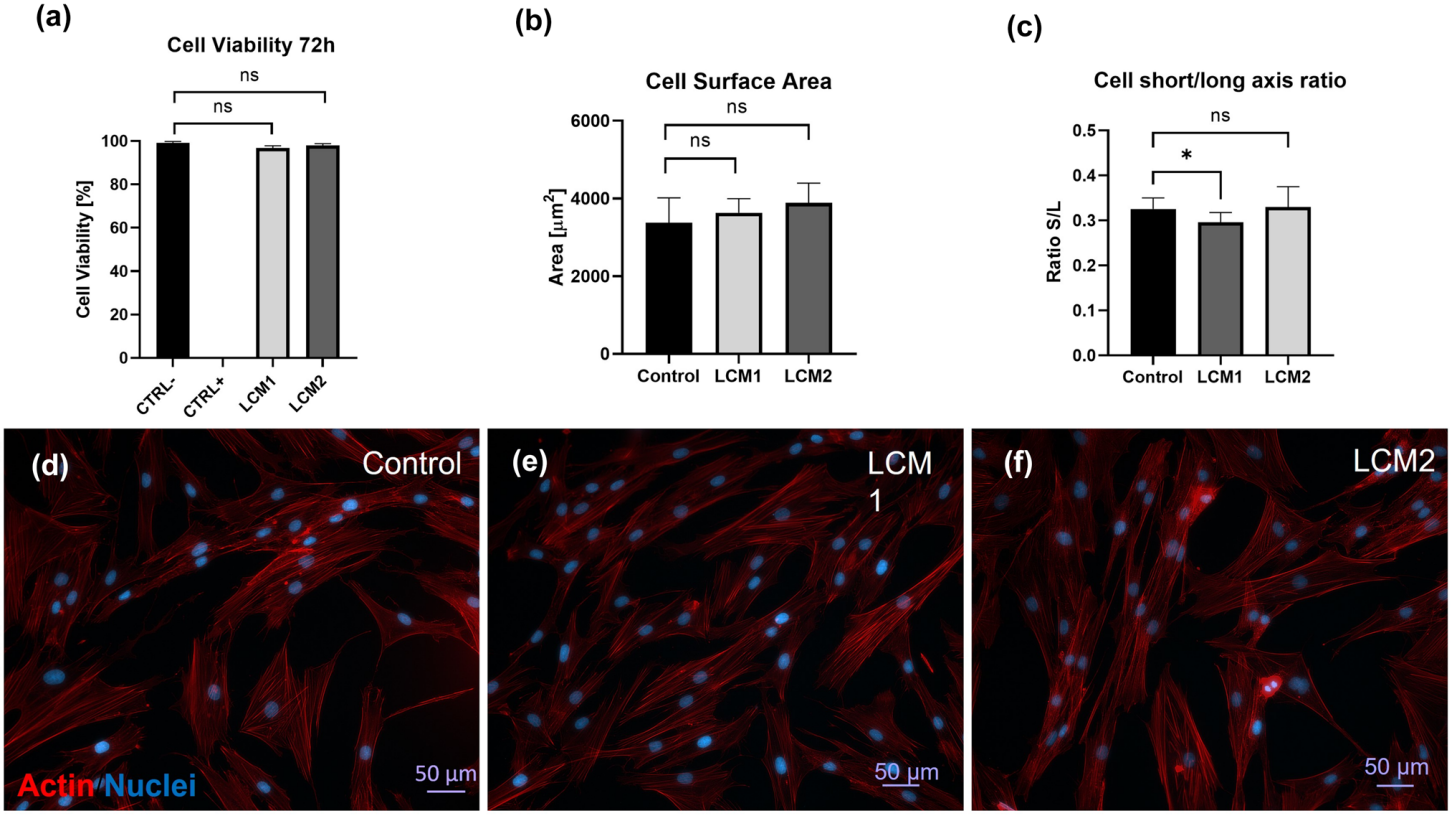
Evaluating cytotoxicity with mesh-conditioned media. **a** Percentage of cell viability after 72 h in contact with EO LCM1- and EO LCM2-conditioned media. **b** Cell surface area and cell elongation (**c**) of HDFs cultured with EO LCM1- and EO LCM2-conditioned media. Immunofluorescence images of HDFs cultured with growth media (**d**), EO LCM1 (**e**) and LCM2 mesh-conditioned media (**f**). *p < 0.05. Scale bar 50 μm

EO LCM1 and LCM2 were incubated with growth media to obtain a mesh-conditioned media to assess cytotoxicity of the meshes that could adversely affect cell viability and morphology. Percentage of cell viability of HDFs in mesh-conditioned media showed no significant difference between EO LCM1, EO LCM2 and the control growth media (Fig. 2a), confirming the safety of EO-treated meshes with HDFs. Qualitative and quantitative evaluations of cellular morphology in contact with EO mesh-conditioned media were also conducted by measuring cellular area and elongation ratio compared to the growth media control. Figure 2b shows no significant difference between the areas of HDFs with EO mesh-conditioned media and the control, while HDFs cultured in EO LCM1 mesh-conditioned media exhibited a more spindle-like shape compared to the control and the EO LCM2-conditioned media, with a statistically significant difference (*p* < 0.05) (Fig. 2c). These findings are confirmed by observing immunofluorescence images of HDFs cultured in the different conditions (Fig. 2d–f).

**Fig. 3:**
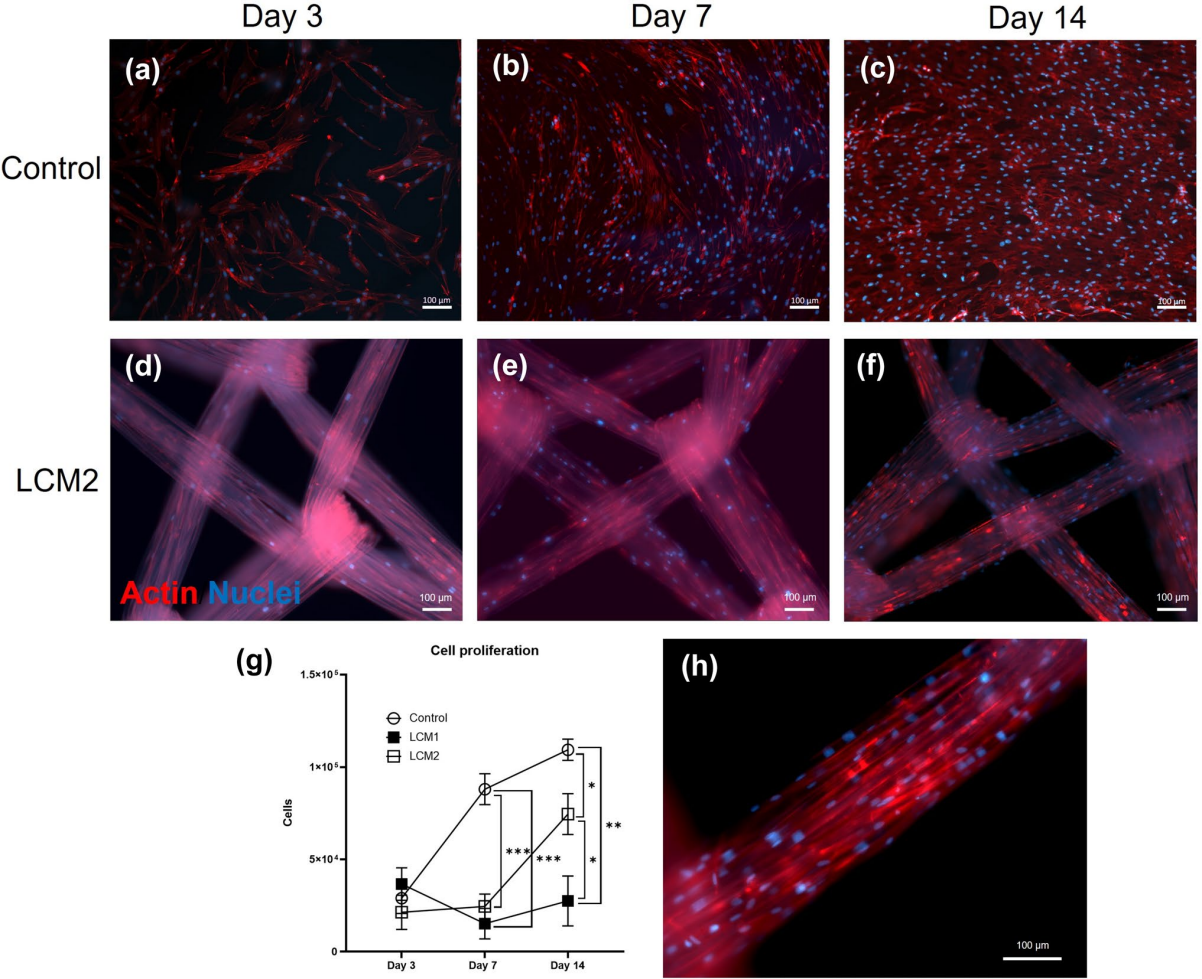
Proliferation and attachment of HDFs on EO meshes. Immunofluorescence images of HDFs cultured on plasticware (control) at day 3 (**a**), day 7 (**b**) and day 14 (**c**) and on EO LCM2 at day 3 (**d**), day 7 (**e**) and day 14 (**f**). **g** Proliferation curve of HDFs on control plastic and EO meshes. **h** Immunofluorescence image of HDFs on LCM2 at day 14 at higher magnification. Scale bar 100 μm. *p < 0.05, **p < 0.01, ***p < 0.001

After confirming that EO-treated meshes do not release cytotoxic components to impair cellular viability, HDFs were seeded directly on the EO-sterilised meshes to verify whether meshes represented a suitable environment for the growth of cells, compared to commonly used tissue culture plasticware. Figure 3a–f shows representative immunofluorescence images of HDFs at day 3, 7 and 14 on EO-treated LCM2. Images for LCM1 meshes are not shown because, although the actin stain worked, the blue DAPI stain created a strong auto-florescence which prevented the visualisation of cells nuclei on the mesh, and therefore did not provide valid information on the attachment. Qualitative observations of HDFs proliferating on that meshes showed that cells had the tendency to grow along the fibrils of the multifilament structure of the mesh fibres, attaching on the surface of the filament, but also filling the inter-filament spaces.

Proliferation of HDFs was evaluated through measurements of metabolic activity on EO-sterilised LCM1 and LCM2 at 3, 7 and 14 days. On day 3, proliferation of HDFs on EO LCM1, EO LCM2 and control was similar, as they recorded comparable values between 2 × 10^4^ and 3.6 × 10^4^ cells per sample. On day 7, the control sample showed a significantly higher number of cells compared to both meshes, with just below 10^6^ cells for the control sample compared to values around 2.5 × 10^4^ for LCM1 and LCM2 (*p* < 0.001). The values at day 7 showed a slow increase in the growth for EO LCM2 and a steeper decrease for EO LCM1. After 2 weeks of culture, the proliferation of HDFs on EO LCM2 significantly increased to approximately 5 × 10^5^ cells, which is still lower than the control sample (now above 10^6^ cells per sample, *p* < 0.05). However, proliferation on EO LCM1 showed little increase compared to the previous time points and the control, as at day 14 the number of cells recorded is similar to day 7, around 2.5 × 10^4^ cells, significantly lower than the control and EO LCM2 (*p* < 0.01 and *p* < 0.05 compared to control and LCM2, respectively).

## Discussion

In this study, we evaluated the effects of EO sterilisation on the mechanical properties and biocompatibility of two UK-sourced LCM, with mechanical properties similar to those of LCM used in LIC. We demonstrated that, despite mechanical properties undergoing a statistically significant change, they remain biologically similar to native tissue. In terms of biocompatibility, LCM2, which was of polyester material, showed no toxic effects and appeared to be a better substrate for HDFs proliferation, compared to LCM1 which was nylon. LCM2 may therefore be considered for human soft tissue reconstruction as a cost-effective intervention.

Low-cost meshes have been adopted as cheaper alternatives to CM in LIC for almost two decades, with numerous studies demonstrating their safety and their likewise surgical outcomes in hernia repair [2, 4, 6, 16]. Despite being evidently cheaper, with each EO-sterilised LCM costing less than £0.30, UK and other high-income countries are still sceptical to the adoption of this frugal innovation in their healthcare systems [17]. Regulatory barriers on the sterilisation methods and the biassed perception of reverse innovation are among the main reasons for this reluctance [18]. Moreover, the high variation in material types and lack of pre-clinical characterisation reinforce this hesitation, as this is an important step to evaluate mesh properties and predict the changes on material structure and properties post implantation.

Regarding in vitro evaluations of LCM, few studies have considered the biocompatibility of LCM towards cells. Sanders et al. compared the adherence of bacteria on autoclaved LCM and commercially available meshes and found no significant difference between the two types, confirming their safety for human implantation [19]. Our previous study evaluated the in vitro biocompatibility of LCM using HDFs and reported high proliferation and attachment to LCM, which is now additionally confirmed on EO-sterilised LCM [20].

Most studies that implanted LCM steam-sterilised at 121 °C for 15 min were found to be more cost-effective than EO sterilisation [9, 21]. However, UK guidelines for medical devices recommend sterilisation at a higher temperature of 134 °C for 3 min but these higher temperatures have shown to alter the integrity of these meshes and these materials undergo significant structural and mechanical alterations [9, 15, 22]. Rynio et al. also evaluated the effects of different sterilisation methods, including EO sterilisation, on various materials [23]. Similar to our study, they reported no changes to the ultrastructure of nylon materials. Their analysis of mechanical properties revealed a major decrease following degradation when immersed in a 0.01 M phosphate buffer saline solution for two months. Another study by Cingi et al. revealed a reduction of the break stress post sterilisation using autoclave sterilisation at 121 °C [24]. Serbetci et al. found that the maximum load before rupture of a propylene mesh decreased slightly both after EO and autoclave sterilisation compared to a non-sterilised control [25]. However, even after considering the changes in the mechanical properties of the EO-sterilised meshes, they still possess a break stress value (9.9 ± 2.1 N/cm) comparable to the native posterior rectus sheath (8.5 N/cm), suggesting they could be an improved alternative to CM, which are typically far stronger and stiffer than needed for their intended function [26].

There have been few studies that have evaluated the effects of EO sterilisation on the proliferation of fibroblasts cultured on surgical meshes. Autoclave sterilisation at 121 °C, which is used to sterilise some LCM in the developing world, appears to impact the proliferation of fibroblasts, as reported by Broll et al. [19]. Savaris et al. [27] examined the biocompatibility of polylactic acid films after EO sterilisation with fibroblasts in vitro and after implantation in vivo, without finding any toxic effect in either study. In addition, other studies examining the differences in properties between gamma-irradiation and EO sterilisation on a collagen-coated vicryl mesh revealed that there were no cytotoxic effects on human fibroblasts, as was confirmed in our study [20, 28].

In conclusion, EO-sterilised LCM demonstrated mechanical properties closer to those of the rectus sheath than existing CM. We also demonstrated that cells were able to attach, remain viable and proliferate when cultured in vitro on low-cost polyester meshes, indicating that EO sterilisation did not significantly hinder biocompatibility. Experiments with longer culture times are needed to evaluate whether biocompatibility and proliferation of cells can be sustained. To assess whether EO sterilisation of LCM would be a cost-effective solution to replace expensive CM, we need to perform a detailed cost analysis of the potential savings that could be made by healthcare systems in HIC. Current European technology appraisal pathways are streamlined for most of the medical devices to be fast tracked into clinical practice for the greater benefit of mankind. More larger scale clinical trials are required within Europe to consider LCM as a suitable alternative for abdominal wall hernia repairs.
